# 

**Published:** 1897-08

**Authors:** 


### CONTENTS.

ORIGINAL COMMUNICATIONS. PAGE
The Use of Silver Salts in the Treatment of Root-Canals. By L. P. BETHEL, D.D.S., M.D., Kent, Ohio . . . . . 485
The Technique of a Transplantation. By LOUIS JACK, D.D.S., Philadelphia . . . . . 494
A Description of some English Appliances. By GEORGE F. GRANT, D.M.D., Boston, Mass. 497
Third Set of Teeth. By S. B. PALMER, M.D.S., Syracuse, N. Y. . . . . . 499
Remarks made at the Anniversary Dinner of the American Academy of Dental Science in Boston, November 11, 1896. By JACOB L. WILLIAMS, M.D. . . . . . 502
What is called a Blind Abscess? By DR. S. ALDEN MILLS, New York . . . . . 506
Cleansing Teeth. By ALBERT H. BROCKWAY, M.D.S., Brooklyn, N. Y. . . . . . 507

ABSTRACTS AND TRANSLATIONS.
The New Antiseptics of Dr. Credé: Silver and the Silver Salts, and their Use in Dentistry. By M. HILLE, Dentist, Dresden . . . . . 508
Carbolic Acid as a Disinfectant . . . . . 511

REPORTS OF SOCIETY MEETINGS.
The New York Institute of Stomatology . . . . . 513
Academy of Stomatology . . . . . 526
Harvard Odontological Society . . . . . 530

EDITORIAL.
Dental Nostrums . . . . . 534
Pennsylvania State Dental Society . . . . . 535
The Coagulation Theory . . . . . 536

BIBLIOGRAPHY . . . . . 537

OBITUARY.
Dr. William Henry Smith . . . . . 540

DOMESTIC CORRESPONDENCE.
Reply of Dr. C. N. Peirce . . . . . 540

CURRENT NEWS.
Report of the Committee on Irregularity at the Pennsylvania State Dental Society, Glen Summit, Pa., July 6, 1897 . . . . . 541
A Voluntary Statement by Dr. Joseph Head, which was accepted by the Pennsylvania State Dental Society, July, 1897 . . . . . 542
The Harvard Dental Alumni Association . . . . . 543
Woman's Dental Association of the United States . . . . . 544
Recent Patents . . . . . 545
Postponement of Meeting . . . . . 546
Northern Ohio Dental Society . . . . . 546

SELECTIONS.
A Dangerous Popular Antiseptic . . . . . 547
Lysol to replace Carbolic Acid . . . . . 547
Origin of Giant Cells . . . . . 548

